# Asbestos Exposure and the Mesothelioma Incidence in Poland

**DOI:** 10.3390/ijerph15081741

**Published:** 2018-08-13

**Authors:** Małgorzata Krówczyńska, Ewa Wilk

**Affiliations:** Department of Geoinformatics, Cartography and Remote Sensing, Faculty of Geography and Regional Studies, University of Warsaw, Warsaw 00-927, Poland; ewa.wilk@student.uw.edu.pl

**Keywords:** asbestos, carcinogen, asbestos-containing products, environmental exposure, malignant mesothelioma, spatial distribution

## Abstract

Asbestos is carcinogenic to humans; the exposure to asbestos causes a wide range of diseases. Aim: Malignant mesothelioma (MM) is unique for asbestos exposure. Methods: Based on the physical inventory of asbestos-cement roofing, the social-economic situation of communes, the proximity of asbestos manufacturing plants, the land use data referring to the surface of the built-up area, and the historical data on the annexations, the amount of asbestos-containing products in use was estimated by computing best Random Forest models. Per capita asbestos use is an indicator to compare the state of asbestos use among countries. MM cases in the local administrative units (provinces) were tested by the application of Moran’s I and Getis and Ord statistic. Results: The total amount of asbestos roofing in Poland was estimated at 738,068,000 m^2^ (8.2 million tons). In total there were 28 plants in Poland located in 11 provinces throughout the country. The amount of asbestos-cement roofing in use is correlated primarily with the measurements of asbestos concentration fibers (*r_s_* = 0.597). MM raw morbidity rate was calculated, stratified by province, and classified into five groups with respect to incidence. Hotspots of MM cases are in the southern part of Poland. Conclusions: MM cases are concentrated in the same geographical areas, which may indicate an increasing impact of environmental exposure. The results of the local and global autocorrelation clearly indicate a statistically significant relationship between incidences of MM in provinces. Poland and other Eastern European countries are among countries with low MM incidence rate. Detailed investigation is desirable since the current MM morbidity rate in Poland seems to be underestimated.

## 1. Introduction

The World Health Organization (WHO) has pointed out that asbestos is carcinogenic to humans [1]. The term “asbestos” refers to a group of naturally occurring fibrous serpentine or amphibole minerals [2]. Due to their extraordinary tensile strength, poor heat conduction, and resistance to chemical attack they were broadly used in the industrial production. The global peak of the production of asbestos-containing products was in the 1960s and 1970s, when there were more than 3000 applications in the national economies [3].

It is estimated that 125 million people in the world are exposed to asbestos fibers with reference to occupational and environmental aspect [4]. Occupational exposure is mainly related to work on the extraction of asbestos in mines or with the production of asbestos-containing products, as well as during the dismantling, the repairs and the maintenance of the products used [5]. Environmental exposure to the pathogenic effect of asbestos and asbestiform fibers mainly affects people living near asbestos mines, asbestos manufacturing and processing plants, people living in highly urbanized areas, where asbestos can be a factor initiating cancer [6,7,8,9].

Potential harmfulness of asbestos fiber was mentioned in medical literature in the 1930s [10]. Asbestos became an established carcinogen for bronchogenic carcinoma and mesothelioma, and malignant mesothelioma (MM) became a symbol of occupational disease in in the second half of the 20th century [11]. In 1969 asbestos (actinolite, amosite, anthophyllite, chrysotile, crocidolite, tremolite) was classified by the International Agency for Research on Cancer as being carcinogenic to humans. In 2012 it was stated that minerals containing asbestos in any form should be regarded as carcinogenic to humans [12]. The number of cases of MM depends on the type of asbestos used, and it increases with the use of crocidolite in production [13]. Mesothelioma risk was much higher when exposure included crocidolite or amosite than chrysotile alone [14]. Crocidolite is considered the most potent fiber type with respect to the pathogenesis of mesothelioma [15]. The negative effects of asbestos and asbestiform fibers on human health are a consequence of inhalation of fibers in the air present in any geographical areas, such as, fluoro-edenite fibers, eronite mineral fibers and tremolite mineral fibers [16]. Those fibers can accumulate in the lung tissue, and their negative impact on health is dependent on the degree of the penetration and the number of fibers retained in the lower respiratory tract [17]. The most important feature determining the ability of fibers to induce tumors is their physical dimensions, that is, diameter below 3 μm and a length of more than 5 μm [18]. Environmental Health Criteria No. 203 (Chrysotile Asbestos) was set by the United Nations Environment Program, which states that no threshold has been identified for carcinogenic risks [19]. Exposure to asbestos causes a wide range of diseases, such as asbestosis as well as cancers such as MM and lung cancer [20]. Asbestos and asbestiform mineral fiber causes initial injury to epithelial and mesothelial cells [21,22,23]. The latency period is very long, that is, most of the human diseases occur decades after exposure to asbestos [24]. To diagnose the disease in a phase in which the surgery and radio chemotherapy may be more effective, new markers (e.g., microRNA) are introduced [16,25]. According to estimates made by the WHO, over 107,000 people die each year due to the asbestos-related diseases. Eliminating asbestos-related diseases is particularly targeted at countries still using chrysotile, in addition to exposures arising from historical use of all forms of asbestos [26]. Asbestos dust and fibers constitute the direct cause of MM [27,28,29]. Malignant mesothelioma is a malignant, rare, and very deadly tumor, a neoplasm typically originating in mesothelial cells lining the body’s serous cavities, mainly the pleura and the peritoneum [30,31,32]. The intensity of exposure is a relevant factor in determining the duration of latency periods [33,34]. The latency period of MM is long [35]. The disease has proven exceptionally resistant to chemotherapy, radiotherapy, and surgery. MM has a very aggressive natural history with a median survival of around nine months [36]. Malignant mesothelioma is classified according to the International Statistical Classification of Diseases and Related Health Problems ICD-10 as C.45 [37] and constituted the subject of the undertaken survey as a unique for asbestos exposure.

The WHO indicated that all countries should implement programs aimed at controlling and eliminating carcinogens in occupational and environmental exposure. Among them, asbestos was listed. The cessation of the use of asbestos for production has been identified as one of the key areas of intervention in the field of environmental exposure [26].

Due to the pathogenic nature of asbestos, in 1997, a statutory ban on the production, use and marketing of products containing asbestos was introduced in Poland (in the European Union the ban has been issued since 1 January 2005 by the adoption of the Commission Directive 1999/77/EC of 26 July 1999). In Polish law, asbestos is considered to be a substance posing a particular threat to the environment, which should be used, transported and eliminated with safety precautions; the installations of devices in which asbestos is or was used should be cleaned or disposed of before 31 December 2032 [38]. Considering the amount and the degree of degradation of asbestos-containing products still used and the long latency period of MM, it is expected that the number of cases of MM will increase in the future. Poland and other Eastern European countries are among countries with low incidence of the detection rate of mesothelioma [39]. The cumulative incidence rate of MM in Poland from 1999–2006 was about 0.3 per 100,000 men and 0.1 per 100,000 women [40].

Carlin et al. [41] have stressed that only with a multidisciplinary approach connecting different sources of data on asbestos will there be an improved understanding of the fiber-induced illnesses, with new risk assessment strategies to protect affected communities. Since there is no threshold set for the concentration of asbestos fibers, below which the risk of asbestos-related cancer does not exist, it is assumed that any long-term exposure to asbestos and asbestiform dust may be a promoter or initiator of cancer [27]. The main aim of the undertaken study is to present the current state of asbestos exposure in Poland, in particular including the use of asbestos-containing products, former asbestos manufacturing plants, and the results of measurements of asbestos fiber concentrations in relation to MM cases. The geographical distribution of MM cases and the occurrence of their potential sources are desirable for the detection of MM. Since Poland is perceived as one of the countries with a low MM incidence rate and insufficient data [39], the undertaken study aims to investigate possible sources of data and MM incidence rates in future.

## 2. Materials and Methods

Data on the quantity of asbestos-cement products in use, asbestos manufacturing plants, the concentrations of asbestos fibers in the air, and the number of MM cases in relation to provinces in Poland were used to determine the relationship between asbestos exposure and incidence of MM.

Based on the physical inventory, the social-economic situation of communes, the proximity of asbestos manufacturing plants, the land use data referring to the surface of built-up area, and historical data on annexations, the number of asbestos-containing products in use was estimated. The physical inventory was taken in 160 communes in Poland with the use of printouts of orthophotomap [42]. Each building with asbestos-containing products was marked on the printout and then digitized, leading to the development of a database of buildings with asbestos-cement roofing. The social-economic features referring to demography, income and expenditure of communes, agriculture area, land use, and buildings characteristics were applied [43]. Land use data for the built-up areas were derived from the Soil Sealing Enhancement Project developed by the European Environment Agency [44]. Historical conditions have had great influence on later types of the land use, the architecture and building construction, and the economy in Poland [45]. Data on historical annexations were acquired from the Mosaic Project [46]. The localization of the asbestos manufacturing plants in Poland, the types of asbestos fibers used, and the kinds of asbestos-containing products fabricated were acquired through field survey and in-depth interviews; a geodatabase on the asbestos manufacturing plants was developed [47]. Data on the quantity of the asbestos-containing products in use per province were obtained by computing Random Forest models; a model explaining 72.9% of the variance was subsequently used to prepare the prediction map of the amount of asbestos-cement roofing in Poland. Predictions were performed with the use of predict() function from the Random Forest package for m^2^ per ha of SSL data, and then was converted into the total value of asbestos-cement roofing in each commune [48]. The information regarding measurements of asbestos fibers in the air carried out in 2004–2007, 2010 and 2012–2013 for the Ministry of Economy were used [49]. The total amount of asbestos fiber consumption was then compared to the selected countries to assess the level of MM cases in Poland. Per capita asbestos use is an indicator to compare the state of asbestos use among countries [50].

Incidence of MM was derived from the National Cancer Register [40], and the Amiantus Program dedicated for former employees of asbestos manufacturing plants [51]. Total number of MM cases (registered under C.45 of 10th Revision of International Statistical Classification of Diseases and Related Health Problems) refers to the period of 1999–2013 and is classified by province and gender. Data on MM cases contain information on the total number of cases, the number of cases of particular types of diseases caused by asbestos, the preventive measures taken, and mortality among people exposed to asbestos fibers. All calculations were performed for males, females, and the general population. To investigate the spatial relationship, Tobler’s law was applied, according to which the neighboring areas are more similar in terms of their features than remote areas [52]. Using spatial statistics, MM cases in provinces were tested. Moran’s I autocorrelation coefficient was used to measure the correlation between neighboring observations in provinces, calculated with the following formula [53,54]:

(1) $$\;I=\frac{n\sum_{i=1}^{n}\sum_{j=1}^{n}w_{ij}(x_{i}-\bar{x})\left(x_{j}-\bar{x}\right)}{\left(\sum_{i=1}^{n}\sum_{j=1}^{n}w_{ij}\right)\sum_{i=0}^{n}{\left(x_{i}-\bar{x}\right)}^{2}},\;i\neq j\;$$

where *n* is the number of study areas (provinces), *w_ij_* represents weight matrix of links between *i* object and *j* object (MM frequency or cumulated incidence in *i* or *j* province), *x_i_*, *x_j_* are variables values in *i* and *j* spatial unit (MM prevalence rate) and $\bar{x}$ is the arithmetic mean of the variable for all units. To examine the measure of the level of the mutual grouping of high and low values, global G Getis and Ord statistic was applied [55,56].

(2) $$G=\frac{\sum_{i=1}^{n}\sum_{j=1}^{n}w_{i,j}x_{i}x_{j}}{\sum_{i=1}^{n}\sum_{j=1}^{n}x_{i}x_{j}},i\neq j$$

where *x_i_, x_j_* are variables values in spatial unit *i* and *j*, *w_i_, w_j_* represents weigh of links between unit *i* and unit *j* and *n* are the number of spatial units. The feature that differentiates the research objects was MM raw morbidity rate due to the lack of data on MM cases divided into the age groups, calculated as follows:

(3) $$\mathrm{SS}\;=\;\frac{\sum_{j=1}^{n}k_{j}}{\sum_{j=1}^{n}p_{j}}\;\times \;100,000\;$$

where *n* is the number of years in the analyzed period, *k_j_* is the number of MM incidence among tested population within the given period *i* and *p_j_* represents the number of people in tested population in the middle of period *j*. Statistical measures of Getis-Ord *G_i_* statistics were used to determine the local pattern indicating points of high MM risk [56]:

(4) $$\;Gi\left(d\right)\;=\;\frac{\sum_{j=i}^{n}w_{ij}\left(d\right)x_{j}}{\sum_{j=i}^{n}x_{j}}\;$$

where *w_ij_* is the weight of links between objects *i* and *j* (MM incidence frequency or aggregated number of registered MM cases in *i* or *j* province), *x_j_* is the variable value in the unit *j* and *d* represents the maximum distance within which the clusters are expected to occur. Gi(d) statistic measures the intensity of clustering of high or low values [57]. To identify statistically significant hot spot and cold spot clusters the Local Moran’s I statistic was applied [58]:

(5) $$\;I_{i}=\frac{n^{2}}{\sum_{i}^{\;}\sum_{j}^{\;}ij}\;x\frac{\left(x_{i}-\bar{x}\right)\;\sum_{j}^{\;}\;w_{ij}\;\left(x_{j}-\bar{x}\right)}{\sum_{j}^{\;}{\left(x_{i}-\bar{x}\right)}^{2}}\;i\neq j\;$$

Fixed Distance band method with the calculated threshold distance was used. For each set, that is, female (cases W), male (cases M) and the entire population (cases all), spatial autocorrelation was measured for the determined threshold distance, based on the *Z*-score value. The level of detection of MM in Poland among men, women and the general population was then compared to those registered in selected countries to determine the relationship between per capita asbestos use and the MM incidence rate. Spearman Rank Correlation Coefficient *r_s_* was calculated to determine the association between the amount of asbestos-cement product in use, asbestos fiber concentrations and the MM morbidity rate.

The information on the territorial division of the country together with the borders of provinces was acquired from Polish National Geodetic and Cartographic Documentation Centre [59].

The relational database, adapted to the requirements of the PostgreSQL database, was designed and developed to provide a geospatial analysis. Compliance with the PostgreSQL database structure meets the requirements of the Electronic Spatial Information System [60] in Asbestos Database for the monitoring of the implementation process of the National Program for Asbestos Abatement in Poland [61].

## 3. Results

### 3.1. The Quantity of Asbestos-Cement Products in Use

**Figure 1:**
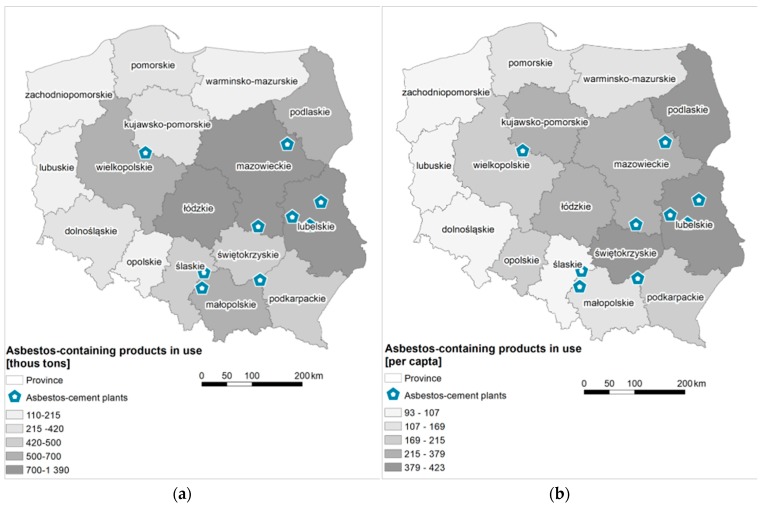
(**a**) The quantity of asbestos-cement roofing in Poland (tons); (**b**) The quantity of asbestos-cement products used in Poland per capita (kg per inhabitant).

The total amount of the asbestos roofing in Poland was estimated at 738,068,000 m^2^ (8.2 million tons). The largest number of asbestos-cement products, that is, 18% of the total estimated amount, falls to Mazowieckie, then to Lubelskie (12%), Łódzkie and Wielkopolskie (each with a 9% share). In Małopolskie, Podlaskie, Świętokrzyskie, Podkarpackie and Śląskie, the share of the estimated number of asbestos-cement products used ranges from 6 to 7%. Kujawsko-Pomorskie province has a 5% share, and 2–3%—Dolnośląskie, Pomorskie, Warmińsko-Mazurskie, Zachodniopomorskie and Opolskie. The lowest share (1%) is characteristic for Lubuskie (Figure 1a). On average, there is 202 kg of asbestos-cement product still in use per one inhabitant of Poland. This indicator has the highest value for Lubelskie—423 kg per person, Podlaskie—416 kg per person and Świętokrzyskie—387 kg per person (Figure 1b). In Łódzkie, Mazowieckie, Podkarpackie and Wielkopolskie amounts to over 200 kg, and in Kujawsko-Pomorskie, Opolskie, Małopolskie, Warmińsko-Mazurskie, Pomorskie, Lubuskie and Zachodniopomorskie—over 100 kg. The smallest number of products per one inhabitant (less than 100 kg) falls on Śląskie and Dolnośląskie.

### 3.2. Asbestos Manufacturing Plants

In Poland, asbestos-containing products were manufactured from raw materials imported from the former Soviet Union (Russia, Lithuania, Kazakhstan, and Belarus), Canada, Italy, Australia, and the UK. Poland does not have natural resources of asbestos mined on an industrial scale. Over 90% of asbestos used in production was chrysotile; and less than 10% constituted crocidolite and amosite, which were used until the 1980s in the manufacturing of pressure pipes [62,63]. According to the Chief Statistical Office data for the period of 1955–1995 the import of asbestos amounted to more than 2,000,000 tons. The peak of import was in the 1970s, when the total amount of asbestos amounted to almost 900,000 tons. In the 1980s, asbestos imports decreased and amounted to over 700,000 tons [64]. It is estimated that more than 75% of asbestos imported into the country has been used for the fabrication of asbestos-cement products, and less than 500,000 tones was the raw material for the manufacture of other asbestos-containing products.

**Figure 2:**
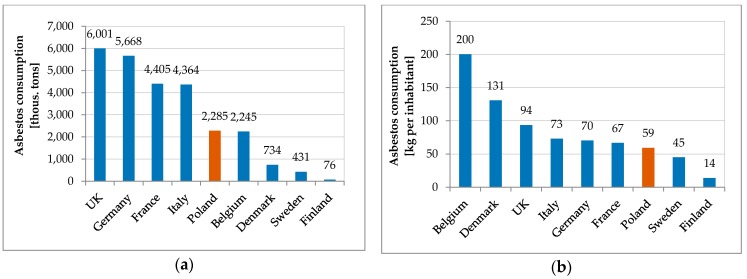
Asbestos consumption in selected European countries. (**a**) Total consumption (thousand tons); (**b**) Total consumption per capita (kg per person).

From 1913–1999 the total amount of asbestos fiber consumption in production was highest in UK. It amounted to over 6,000,000 tons. Germany was the second largest producer of asbestos-containing products with the total consumption amounted to 5668 thousand tons. The level of consumption of asbestos fibers for production purposes in Poland was similar to Belgium, much lower than in the UK, Germany, France, and Italy (Figure 2a) [65,66]. Belgium has the highest total consumption of asbestos fibers per capita at 200 kg per inhabitant. Comparing data on the consumption of asbestos for the production purposes per capita, Poland can be compared to Italy, Germany, and France (Figure 2b).

**Figure 3:**
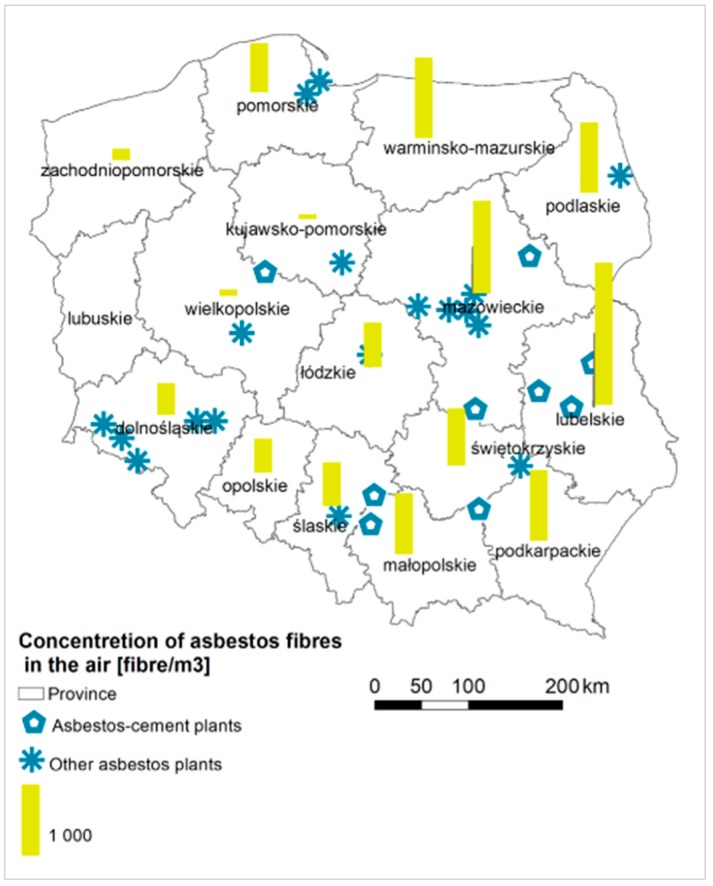
Measurements of concentrations of asbestos fibers in the air in relation to asbestos manufacturing plants in Poland.

In total there were 28 plants that used asbestos in production, of which 10 produced asbestos-cement products [47]. The peak production period was in the 1970s, when more than 50 million m^2^ of corrugated and flat sheets used in construction were produced annually [64]. Asbestos manufacturing plants were in 11 provinces throughout the country (Figure 3). In 1998 the manufacturing process was terminated in all plants [67]. Relatively high concentration of asbestos-cement products in use is observed in the proximity of asbestos-cement manufacturing plants, that it, Małkinia and Wierzbica (Mazowieckie), Lublin (Lubelskie), Szczucin and Trzebinia (Małopolskie), Ogrodzieniec (Śląskie), and Trzemeszno (Wielkopolskie) (Figure 1a).

### 3.3. Concentrations of Asbestos Fibers in the Air

Asbestos fiber concentration measurements were carried out from 2004–2013. They were executed by passing a certain amount of air through a membrane filter (from cellulose esters) with a pore size of 0.8 μm and a diameter of 25 mm by a pump with a controlled volume of the air. The determination of the sampling point, the measurement points, and the air intake were made in accordance with the PN-88/Z-04202.02 standard [49]. In total they were carried out in 1037 communes [49], which constitute 42.4% of Poland’s area, where more than half of the population lives, that is, over 22 million people. There were asbestos fiber concentrations results above 5000 fibers per m^3^ recorded in Lubelskie (3 communes), Łódzkie (3 communes), Małopolskie (1 commune) Warmińsko-Mazurskie (1 commune) and in Mazowieckie (1 commune). The highest average concentration amounts to 8229 fibers per m^3^ (Annopol in Lubelskie). In five provinces (Lubelskie, Mazowieckie, Podlaskie, Podkarpackie, Warmińsko-Mazurskie), the average concentration is about 1000 asbestos fibers per m^3^. The lowest level of asbestos fiber concentration was measured for Kujawsko-Pomorskie, Opolskie and Wielkopolskie (Figure 3). The amount of asbestos-cement roofing in use is correlated primarily with the measurements of asbestos concentration fibers (*r_s_* = 0.597).

### 3.4. Malignant Mesothelioma Cases

The National Cancer Register was developed to improve the quality of epidemiological data on malignant cancers and to enable modern epidemiological studies with the use of information society technologies as a result of the implementation of the Act on Information System in Health Care. Data gathered in the National Cancer Register refer to all provinces and are provided by the provincial medical centers according to the legal regulations. The competence of the National Cancer Registration Office is the collection and processing of data on diagnoses and suspected malignancies, including data necessary to perform the tasks related to public statistics. Reporting of the diseases to the National Cancer Registry is carried out by provincial centers [68]. In Poland, physical examination of MM patients involves standard methods of assessing the chest and the respiratory system. Imaging examinations such as chest X-ray, computer tomography, and magnetic resonance enable precise evaluation of the condition’s extent (infiltration of the chest wall, pericardium, and diaphragm) [69].

**Figure 4:**
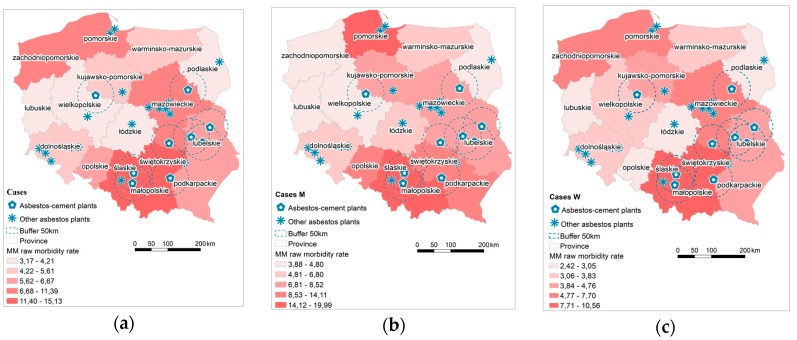
Malignant Mesothelioma (MM) raw morbidity rate (**a**) cases all; (**b**) cases M (men); (**c**) cases W (women).

Based on data derived from the National Cancer Register, MM raw morbidity rate was calculated, stratified by province, and classified into five groups with respect to incidence (Figure 4). Based on the cumulative number of reported MM cases, three provinces (Śląskie, Małopolskie and Świętokrzyskie) were classified with the highest morbidity rate amounting to over 11 cases per 100,000 inhabitants. There were no statistically significant correlations found between the number of asbestos-cement products in use and the number of MM cases.

There is a statistically significant positive autocorrelation of the occurrence of the cumulative MM morbidity rate for the entire population and divided into male and female (Table 1).

**Figure 5:**
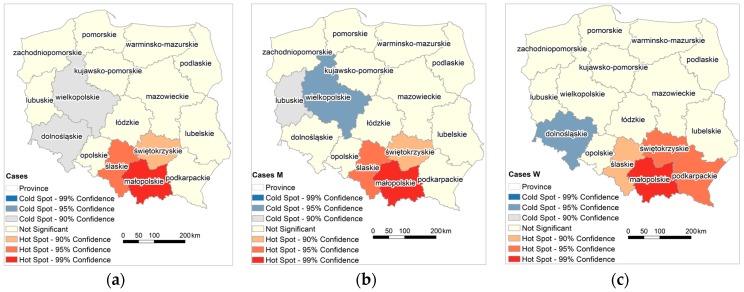
Hot spot analysis of MM morbidity rate in provinces (**a**) cases all; (**b**) cases M; (**c**) cases W.

Hot spots of MM cases are in the southern part of Poland, that is, Śląskie, Małopolskie and Świętokrzyskie; additionally, Podkarpackie appears for female cases (Figure 5). A cold spot was recorded in male population in Wielkopolskie, and in female population in Dolnośląskie.

**Figure 6:**
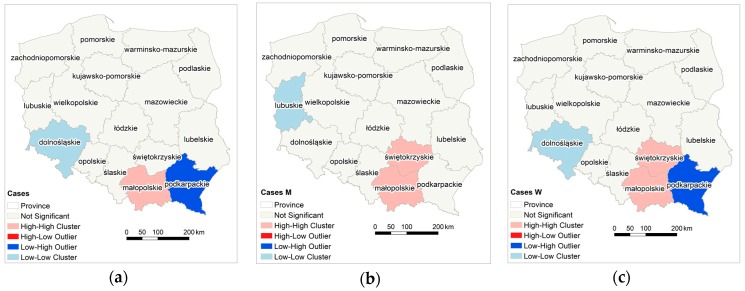
Local autocorrelation of MM morbidity rate in provinces; (**a**) cases all; (**b**) cases M; (**c**) cases W.

In southern Poland, clusters of high values of the MM morbidity rate have been determined; in Małopolskie for the entire population and in Świętokrzyskie for female and male population (Figure 6). In case of female population there is a clear boundary between the eastern part of Małopolskie and Świętokrzyskie appointed by the Low-High province (Podkarpackie). Moving west, there are provinces with low values of the MM morbidity rate, also surrounded by provinces with low values of Low-Low MM morbidity rate (Dolnośląskie for the entire and female population, and Lubuskie for male population).

**Figure 7:**
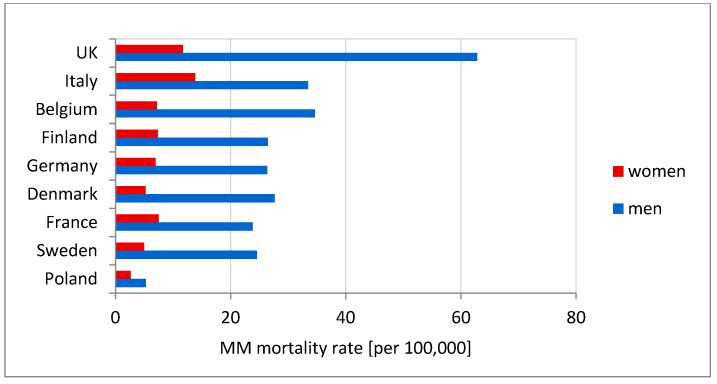
MM mortality rates in selected European countries.

MM mortality rates were derived from the WHO Mortality Database [70] and differ among countries. The highest mortality rate is denoted for the UK, Italy, and Belgium. Incidence of MM in men and women differs significantly (Figure 7). Compared to Western European countries, Poland has the lowest mortality rate.

**Figure 8:**
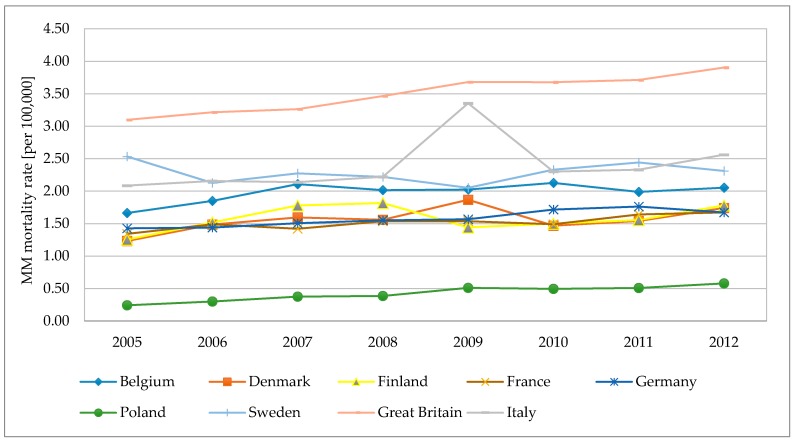
MM annual mortality rates in selected European countries in 2005–2012.

Moreover, comparing annual data on MM mortality rates [70] (Figure 8), the highest and still growing MM mortality rate is observed for Great Britain. In Italy there was a peak observed in 2009. In Poland, the MM rate is at the lowest level, and a growing trend is denoted.

## 4. Discussion

Analyzing MM incidence in Poland and the factors affecting asbestos exposure is aimed at pointing out the circumstances in which the development of the disease begins, which may be the promoter of its development, such as, environmental factors [71,72]. Poland has low morbidity or detectability rate of MM in comparison with other European countries. The number of detected men’s cases in 1999–2013 is five times lower than in Italy, Germany, and Sweden (Figure 7), while the number of women’s cases is half that in Sweden and Denmark, and three times lower than in other Western European countries. There is a link between historical asbestos consumption and MM morbidity rate; 1 kg per capita per year of asbestos use corresponds to 2.4- and 1.6-fold increases in MM death among men and women, respectively [73]. Per capita asbestos use, in terms of consumption, is a substitute measure for general exposure level, to estimate consequent health burdens at the national levels [74]. Comparing per capita asbestos use, it may be concluded that Poland is relatively similar to Italy, Germany, France, and Sweden [65,66] (Figure 2b). Considering the MM morbidity rate in comparison to per capita asbestos use, Poland has 4-fold lower MM detection than other European countries (Figure 7) and is perceived as a country with the lowest incidence MM registered rates [39,40,70]. It must be stressed that the peak of asbestos consumption in Poland in comparison to the UK, Belgium and France shows a tendency to shift by a decade [65,66]. This may be the result of an underestimation of MM incidence in Poland, which might be recognized in 10 years. From an epidemiological point of view, regions of interest are where incidence of MM is higher. The results of the local and the global autocorrelation clearly indicate a statistically significant relationship between incidences of MM in particular regions of Poland (Figure 5). High values of the MM morbidity rate in provinces concentrate around asbestos-cement manufacturing plans. Their proximity has an impact on the number of MM cases [75]. The highest value of MM morbidity rate was recorded in Małopolskie and Śląskie. In addition, in Świętokrzyskie for women cases there is the highest morbidity rate denoted. In this province, in the commune of Szczucin in 1959–1999, the Factory of Asbestos-Cement Products was operating, where about 1000 different products, mainly asbestos-cement roofing, asbestos-cement pipes with large diameters, for the production of which crocidolite was used [76]. Moreover, in Szczucin commune for about 30 years, asbestos-cement waste was used for the hardening of roads, courtyards, and sports facilities [77]. The results of increased morbidity in women population in this region may indicate asbestos environmental exposure. All asbestos manufacturing plants have ceased production since 1998. Since the latency period of MM is quite long, there may be an underestimation of MM cases in the proximity of former asbestos manufacturing plants [78], not only in terms of occupational exposure, but also environmental exposure, in particular when women are concerned. The percentage of cases of mesothelioma among women is lower than among men (Figure 4) [78,79]. This may be because men more often than women worked in the asbestos industry [80,81]. Correlations between MM incidence ratio in male and female population for Poland amounted to 0.83, in comparison to the incidence ratio in France of 0.80 [79]. There were also correlations found between geographic patterns of MM cases among men and women (Figure 5 and Figure 6). MM cases, in men and women, as well as in the general population are concentrated on the same geographical areas, which may indicate an increasing impact of environmental exposure [79].

Maule et al. [82] found a comprehensive estimate of asbestos domestic exposure to asbestos, to include the information of presence and use of asbestos materials in the house and its proximities, as well as the distance from asbestos manufacturing plants. There was a positive correlation found between the amount of asbestos-cement product in use and the measurements of asbestos fiber concentrations in the air in the provinces in Poland, and partially with the proximity of asbestos-cement plants and the air pollution. It was examined that the strength of the effect of pollution from other sources on the general population could reach up to 1/3 of the risk of asbestos industry workers [82].

For over 20 years in Poland, since the production of asbestos-containing products has been banned, the population is exposed to environmental asbestos. Spearman’s correlation coefficient rank was calculated for the number of asbestos-cement products per capita and the MM incidence ratio; there was no statistically significant correlation found. This may be caused by the long latency period, which was not revealed by environmental exposure. The risk of disease rises with the increase of the amount of dust in the air [83]. The smallest air pollution is denoted in the eastern part of Poland, where the largest number of asbestos-cement products is still in use. Areas with the highest risk of air pollution with substances constituting health risks for people are in Śląskie, Małopolskie, and Świętokrzyskie [84].

Driece et al. [85] examined asbestos exposure by incorporating the following factors: the distance to plant, the sites with the asbestos-containing products in use and its amount, the asbestos waste, the measurements of asbestos fiber concentrations in the air. Asbestos products in use and asbestos waste may result in long-term exposure to asbestos of about 2000 fibers/m^3^ and these concentrations may lead to a couple of cases of MM each year [85]. The measurements taken in Poland revealed that the average fiber concentration is approximately 700 fibers/m^3^ (Figure 3). Taking into the account the period of the safe use of asbestos-cement roofing for over 30 years [62], it is expected that in the near future the asbestos fiber concentrations will rise due to the deterioration of asbestos-containing products. There is a positive correlation found between the number of asbestos-cement products in use and the measurements of asbestos fiber concentrations in the air, which may act as an indicator of environmental asbestos exposure.

There is no adequate data on asbestos-containing products in use to be compared between countries. For modeling asbestos exposure, researchers examine areas in the proximity of asbestos factories [82], establish the links between MM cases based on the total asbestos consumption at country level [73,74,86,87] or present the geographical distribution of the MM cases [79], in particular linked to the asbestos factories [78]. However, possible factors influencing asbestos exposure are determined, such as: the number of asbestos-cement products, the asbestos fiber consumption in production, the asbestos manufacturing plants, and the asbestos fiber concentrations in the air. Considering the aforementioned factors, a detailed study on an underestimation of the MM cases in Poland should be undertaken to find all relevant factors that may influence the MM morbidity rate for modeling the mesothelioma risk associated with environmental asbestos exposure in the future. It should be considered also that the health policy based on the best estimates of asbestos exposure risk detects the MM cases with least delay. Incidence of the MM cases in women may act as an indicator of environmental asbestos exposure [88]. Since there is a time lag between asbestos exposure and the mesothelioma occurrence which may exceed 40 years [86], all potential factors and methods should be considered for the geographical distribution and the modeling the number of the MM cases to implement the health policy measures to detect the potential clusters of MM in Poland to protect the affected communities, despite the fact that currently there is an underestimation of incidence of MM in comparison with Western European countries. As the asbestos production shifts to developing countries, knowing the factors that are influencing environmental exposure may lead to a global asbestos ban that prevents asbestos exposure worldwide.

## 5. Conclusions

The study gathered data on the MM cases in Poland and the factors potentially affecting the disease in relation to province. The database containing the number of asbestos-cement products in use, the details on asbestos manufacturing plants, and the results of the measurements of asbestos fiber concentrations in the air was developed to assess the possible factors influencing environmental asbestos exposure. The spatial and geographic patterns of MM morbidity rate for women, men and the entire population were determined. Geographical patterns of incidence for men and women are consistent. The number of cases of women is lower than that one of men, respectively 36% and 64%. Data regarding MM cases for both men and women, and the general population is concentrated in the same geographical areas, which may indicate the increasing impact of environmental exposure. The hotspots of MM cases in all examined cases are in the southern part of Poland, that is, Śląskie, Małopolskie, and Świętokrzyskie, which may be connected with air pollution, since this is an area that poses a health risk to people, the proximity to asbestos manufacturing plants, and the number of asbestos-cement products in use. Due to the lack of the results regarding the amount of the currently used asbestos-containing products in other countries, it is difficult to assess the number of the cases of MM in the future and to assess the currently diagnosed number of cases as high or low. All possible and potential factors affecting the future MM morbidity rate were taken into the account, that is, the number of asbestos-cement products in use, the proximity, the type of production in asbestos manufacturing plants, and the results of the measurements of asbestos fibers in the air. Detailed investigation is needed since the current MM morbidity rate in Poland seems to be underestimated, particularly when compared to other Western European countries in terms of the asbestos use per capita. However, the early detection of MM is not leading to the increase of the period of survival, studies on the molecular pathogenesis and the immunological tumor microenvironment of MM, regarding the role of BRCA1 associated protein 1 (BAP1) and the expression programmed death receptor ligand 1 (PD-L1), are highlighting new and potential therapeutic strategies [89].

Most researchers compare the consumption of asbestos fibers per capita between selected countries and combine historic consumption of asbestos fibers in production with the number of MM cases, indicating that the increase in production was associated with the increase in the number of MM cases. Most of these studies were directly linked to occupational exposure. At present, environmental exposure will have increasing impact on incidence of MM. The best measure to assess its impact seems to be monitoring of concentrations of asbestos fibers in air. Due to the high costs associated with performing such measurements, few countries take up such challenges nationwide. In Poland in the period of 2004–2013, measurements were performed in 190 communes; the analysis shows that there is correlation between the size of concentrations of asbestos fibers in the air and the number of asbestos-cement products used. The proper direction for future study is to undertake research on modeling the quantity of asbestos products used to estimate the number of MM cases, indicating the potential places of occurrence.

## Figures and Tables

**Table 1:** Global Moran’s I statistics for the cumulative MM morbidity rate in Poland.

MM Morbidity Rate	Moran’s I	E (I) s ^1^	Var (I) ^2^	*Z*-Score ^3^	*P*
Entire Population	0.287	−0.066	0.02	2166	0.03
Man	0.260	−0.066	0.02	2012	0.04
Women	0.269	−0.066	0.02	2012	0.03

^1^ Expected index value; ^2^ theoretical variance; ^3^ standard normal deviation $Z=\frac{I_{i}-\bar{E}\left(I_{i}\right)}{\sqrt{VAR\left(I_{i}\right)}}$; *P* < 0.05 is considered significant.
